# Fecal-Oral Transmission of SARS-CoV-2 In Children: is it Time to Change Our Approach?

**DOI:** 10.1097/INF.0000000000002704

**Published:** 2020-04-16

**Authors:** Daniele Donà, Chiara Minotti, Paola Costenaro, Liviana Da Dalt, Carlo Giaquinto

**Affiliations:** From the * Division of Pediatric Infectious Diseases, Department of Woman’s and Child’s Health; † Department of Woman’s and Child’s Health; ‡ Pediatric Emergency Department, University of Padua, Padua, Italy.

**Keywords:** COVID-19, children, gastrointestinal, rectal swab

## Abstract

Starting from 2 pediatric cases of COVID-19, with confirmation at nasopharyngeal and rectal swabs, we considered the lesson learnt from previous Coronavirus epidemics and reviewed evidence on the current outbreak. Surveillance with rectal swabs might be extended to infants and children, for the implications for household contacts and isolation timing.

In Italy, we have been currently experiencing the effects of severe acute respiratory syndrome (SARS)-CoV-2 global spread, started at the end of 2019 in Wuhan. Findings from the impact of COVID-19 pandemic on pediatric patients are few. However, children seem to present more often gastrointestinal symptoms than adults, with reported vomiting, abdominal pain and diarrhea.^1^ Additionally viral shedding has also been reported in children without gastrointestinal symptoms and has been linked to a possible long-term fecal-oral transmission.^2^

In March 2020, 2 infants with SARS-CoV-2 infection were admitted to our Pediatrics Department. The first one, a 5-month-old boy, presented respiratory and gastrointestinal symptoms with diarrhea. Nasopharyngeal and rectal swabs were taken, with a positive result. He was discharged in mandatory home isolation, afebrile and asymptomatic. The second one, 2-months-old, presented only mild respiratory symptoms. After COVID-19 infection was confirmed with nasopharyngeal swab, despite the absence of gastrointestinal symptoms and based on the findings of the previous case, he also underwent a rectal swab, that tested positive for SARS-CoV-2 on day 3 from onset.

According to the recommended dispositions provided by Italian Ministry of Health, the follow-up of these patients, to avoid contagion and uncontrolled spread of the disease, implies mandatory strict home isolation until the finding of 2 subsequent negative results at nasopharyngeal swab. However, there are no current official disposals concerning rectal swabs, for further investigations, with no implications on isolation timing.

## STATE OF THE ART AND FUTURE DIRECTIONS FOR SARS-COV-2 STARTING FROM LESSONS LEARNT FROM PREVIOUS EPIDEMICS

During the SARS outbreak in 2002 to 2003, there were reports of viral RNA being found in fecal samples, occasionally even after 30 days after symptoms onset, determining a risk for the stools to become a source of contamination of airdrops and several environmental surfaces.^3^ In children, SARS-CoV infection was associated to gastrointestinal symptoms, but there is no evidence of rectal swabs being performed for diagnosis and further surveillance.

In Middle East Respiratory Syndrome-CoV epidemic in 2012, there was proof of viral RNA detection in fecal specimens in adults, but there were no data about surveillance in children, despite reported occurrence of gastrointestinal symptoms.^1^

As for COVID-19 outbreak, the most relevant international evidence is reported in Table 1. Zhang et al^4^ reported that SARS-CoV-2 RNA was found in stool specimens and rectal swabs, often with a higher number of positivities than oral samples in a later phase of disease. These findings might suggest that, if feasible, “non-infectivity” should not rely only on negativity of oral swabs, as the virus might still be shed in the body fluids.^4^

**TABLE 1.**
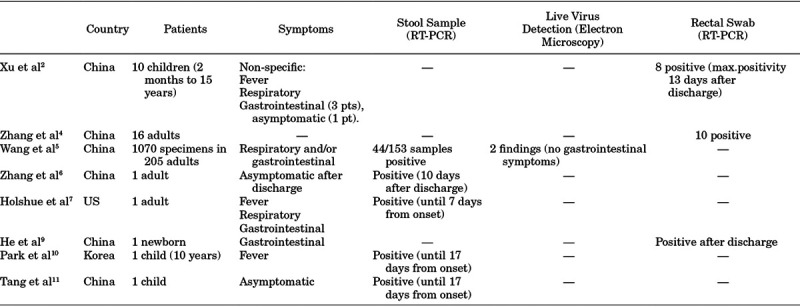
Evidence of SARS-CoV-2 Detection in Stools and Rectal Swabs in Adults and Children

Wang et al^5^ reported detection of viral RNA in respiratory tract swabs and stools, with 44/153 positive fecal samples. Four positive fecal samples showed high copy numbers with a mean cycle threshold (Ct) value of 31 4 (<2.6 × 10^4^ copies/mL). Live virus was found in feces of patients without diarrhea, suggesting a systemic infection. The spread of the virus by also non-respiratory routes might justify the rapid diffusion of infection and testing of samples from multiple sites may be useful to enhance sensitivity and decrease false-negative results.

Zhang et al^6^ reported an adult case that presented after initial negativization positive sputum and fecal samples, with the latter still testing weakly positive 10 days after discharge. The hospital revised criteria for discharge, requiring 2 consecutive negative samples from the respiratory tract and stool alike.

Holshue et al^7^ published the first adult case in the US, showing high viral loads in nasopharyngeal specimens at onset, with a tendency to decrease following disease course, with increasing Ct values. In addition, viral detection in stool samples occurred at 7 days from onset.

An initial concern was the sensitivity and specificity of SARS-CoV-2 PCR tested in stools. Recently, the same level of accuracy has been demonstrated for stool samples and pharyngeal swabs, regardless of symptoms and with no correlation to disease severity.^8^

Concerning the pediatric population, evidence on the use of rectal swabs or viral detection in stools is reported in Table 1. Xu et al^2^ reported ten pediatric PCR-confirmed cases of SARS-CoV-2 infection, all with non-specific symptoms. The children’s pattern of viral shedding was monitored with subsequent nasopharyngeal and rectal swab samples: 8 patients showed persistent positivity on rectal swabs, with 2 of them remaining positive for up to 13 days after discharge, also after nasopharyngeal swabs had turned negative. Viral loads showed that shedding from the gastrointestinal tract may be higher and more long-lasting compared with the respiratory tract.

As reviewed by He et al,^9^ a newborn presenting vomiting and diarrhea as first symptoms was tested positive for SARS-CoV-2, showing negativization of pharyngeal swab after treatment but a persistently positive rectal swab.

Park et al^10^ reported a pediatric case with positive nasopharynx, throat and feces samples on admission. By day 16 from symptoms onset, throat swab samples had turned negative, while on day 17, viral RNA was still found on feces and with weak positivity on nasopharyngeal samples.

Last, Tang et al^11^ showed how an asymptomatic child presented a positive fecal sample for up to 17 days since the last exposure, with reported negative samples from the respiratory tract.

Droplets are the main human-to-human mechanism of transmission of SARS-CoV-2, but fecal shedding with environmental contamination may play an important role in viral spread. As pointed out by Li et al,^12^ there is a great number of infections not being documented, especially in paucisymptomatic or asymptomatic individuals, which may have helped the fast diffusion of the virus.^12^ The clinical pattern of disease presentation among children may have facilitated viral dissemination. Moreover, there is evidence supporting viral viability in environmental settings that may predispose fecal-oral transmission, with recent evidence supporting that SARS-CoV-2 can remain viable in aerosols up to 3 hours, and for 72 hours on solid surfaces, similarly to SARS-CoV.^13^

Not only the importance of correct hand hygiene should be encouraged by every mean, but severe measures must also be observed handling the feces of infected patients, and sewage from hospitals requires proper disinfection.

Current evidence brings concerns on excluding SARS-CoV-2 infection by single time point nasopharyngeal swabs, with sensitivity being dependent on the test’s characteristics and technique of collection of the samples, with increasing data hinting at fecal transmission as an important alternative route.

## CONCLUSIONS

Since gastrointestinal symptoms seem to be more frequently reported in children than adults, and in view of current evidence of fecal shedding, there are implications for every child being admitted or home-isolated, and for household contacts. Indeed, rectal swabs should be considered especially in children for diagnosis as well as to better define the duration of isolation, along with findings from nasopharyngeal swabs.

Further evidence on gastrointestinal involvement and excretion of SARS-CoV-2 in stools is necessary to confirm fecal viral loads regardless of enteric symptoms, and to better explore viral RNA detection in the early incubation or late convalescence stages.

A negativity in both nasopharyngeal and stool samples might be considered as a standard requirement for cessation of mandatory isolation, especially in those settings where there is a risk of infecting vulnerable populations (eg, retirement homes).

## ACKNOWLEDGMENTS

All authors have made substantial contributions to the conception, design, collection, and interpretation of data for this article, drafted the manuscript, revised it critically for content, and approved the final version.
